# Translational potential of γδ T cells in hematologic diseases: from immunobiology to therapeutic innovation

**DOI:** 10.3389/fimmu.2026.1775981

**Published:** 2026-04-24

**Authors:** Xiaokuan Zhou, Mengqi Zhai, Zhe Zhang, Guojun Zhang

**Affiliations:** 1 Department of Hematology, The Fourth Affiliated Hospital of China Medical University, Shenyang, Liaoning, China; 2 Department of Urology, The First Affiliated Hospital of China Medical University, Shenyang, Liaoning, China

**Keywords:** CAR-γδ T cell therapy, cytokine plasticity, hematologic malignancies, translational barriers, γδ T cells

## Abstract

Hematologic disorders, including malignant and autoimmune conditions, present persistent clinical challenges characterized by relapse, treatment resistance, and profound immune dysregulation. While conventional immunotherapies have advanced, their efficacy is frequently limited by HLA downregulation and effector T cell exhaustion. In this context, γδ T cells offer a promising therapeutic alternative. Recognizing antigens independently of MHC restriction, γδ T cells possess intrinsic tissue-homing capabilities and exhibit dual cytotoxic and immunoregulatory functions. These properties make them highly suitable candidates for allogeneic, “off-the-shelf” cellular therapies where αβ T cells face alloreactive limitations. This review systematically synthesizes the immunobiology of γδ T cells, exploring the functional heterogeneity of specific subsets and their regulation within the tumor microenvironment (TME). We critically evaluate recent preclinical and clinical evidence supporting adoptive transfer, CAR-γδ T strategies, and combination regimens across acute leukemias, lymphomas, multiple myeloma, and immune cytopenias. Furthermore, we address critical translational barriers—including *in vivo* persistence, subset exhaustion, and manufacturing variability—and discuss rational engineering strategies, metabolic preconditioning, and epigenetic modulation as solutions. Ultimately, advancing γδ T cell therapies requires overcoming these hurdles to transition them effectively from the bench to mainstream clinical practice.

## Introduction

Hematologic disorders, both benign and malignant, represent a heavy and expanding burden on global health. Non-malignant conditions such as autoimmune hemolytic anemia (AIHA), severe aplastic anemia (SAA), and immune thrombocytopenia (ITP) are far from harmless. One-year mortality of severe aplastic anemia reaches 30-40%, while autoimmune hemolytic anemia triples the risk of thromboembolism, with death in nearly one quarter of cases (1–3). Malignant blood system diseases are in the same situation. In elderly patients with acute myeloid leukemia (AML), the five-year survival rate falls below 20%, and relapse brings a median overall survival (OS) of only six to eight months (4, 5). Relapse itself is the familiar outcome across acute lymphoblastic leukemia (ALL), chronic lymphocytic leukemia (CLL), and multiple myeloma (MM), most severe in those with adverse genetic profiles (6–8). Even as targeted drugs and immune checkpoint inhibitors emerge, access remains profoundly unequal: in low- and middle-income countries, fewer than five percent of eligible patients undergo allogeneic stem cell transplantation (9–11). Resistance to therapy, immune evasion through HLA loss, relentless relapse, and toxic side effects all make clear the need for new immune-based treatments-therapies beyond the confines of MHC, designed for universal use.

γδ T cells represent a distinct group of T lymphocytes, positioned between innate and adaptive immunity (12, 13). Conventional αβ T cells, in contrast, depend on MHC-restricted recognition and fall neatly into CD4+ helper and CD8+ cytotoxic categories (12, 14). γδ T cells do not follow this rule. They detect a wide variety of antigens-stress ligands, phosphoantigens, and lipids-without the need for MHC. This feature provides a potential therapeutic advantage in hematologic malignancies with HLA downregulation: it gives them an advantage against hematologic malignancies that escape immune attack by reducing HLA expression (15–17). Their behavior is fast and decisive. They kill directly, release pro-inflammatory cytokines, and move with accuracy to tissues such as the bone marrow, the site where many blood disorders begin (18, 19). Their biology looks almost paradoxical. They are innate-like, yet they adapt. They can be modified with tools like CAR constructs (20–22). Crucially, their MHC-independent recognition intrinsically bypasses alloreactivity and the risk of graft-versus-host disease (GVHD). This supports the potential of γδ T cells as a promising platform for developing universal, allogeneic ‘off-the-shelf’ immunotherapies, eliminating the need for patient-specific HLA matching. Just as important, they play both sides of the immune system-driving activation when needed and imposing regulation when required. That dual role allows them to address malignancy and autoimmunity alike, making them unusually versatile across the spectrum of disease.

This review offers a translational perspective on γδ T cell biology and their therapeutic promise in hematologic disease, with attention to unmet needs, subset-specific mechanisms, and evidence from early clinical trials. We bring together current findings on their cytotoxic and regulatory roles, approaches to engineering, and applications across leukemia, lymphoma, multiple myeloma, and autoimmune cytopenias. Particular focus is given to obstacles that stand in the way-functional exhaustion, inconsistency of expansion, and donor diversity-and to strategies that may overcome them, such as cytokine conditioning, bispecific targeting, and GMP-compliant manufacturing. The purpose is simple but far-reaching: to connect immunobiology with clinical necessity, and to suggest how γδ T cells may be developed into a new platform of immunotherapy for patients with refractory or high-risk blood disorders.

## Overview and translational implications of γδ T cells

### Overview of the T cell system: a prelude to γδ T cell identity

**Figure 1:**
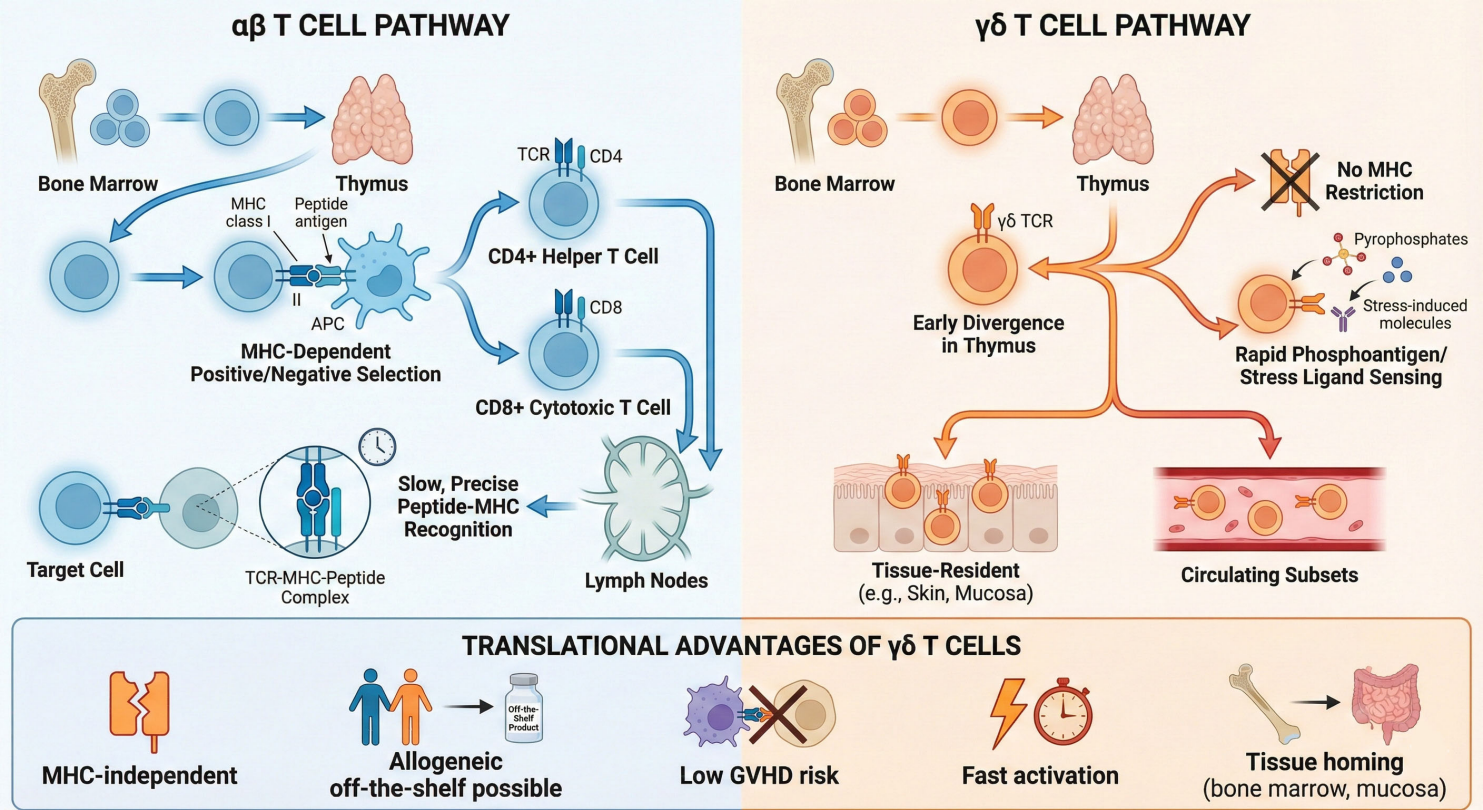
Comparative positioning of αβ and γδ T cells within the immune system and their translational implications. Conventional αβ T cells require classical antigen-presenting cells (APCs) to process and present specific peptide antigens via Major Histocompatibility Complex (MHC) molecules. This recognition by the αβ T-cell receptor (TCR) is heavily restricted by human leukocyte antigen (HLA) matching, which drives a slower, adaptive immune response and carries a high risk of alloreactivity and graft-versus-host disease (GVHD) in allogeneic transplant settings. In stark contrast, γδ T cells operate independently of MHC restriction. The γδ TCR, alongside innate co-receptors such as NKG2D, directly recognizes non-peptidic antigens (e.g., phosphoantigens) and stress-induced ligands (e.g., MICA/B) expressed on the surface of transformed or infected cells. This innate-like, rapid recognition mechanism intrinsically bypasses the need for patient-specific HLA matching and minimizes alloreactive GVHD risk, firmly establishing γδ T cells as ideal candidates for the development of universal, “off-the-shelf” allogeneic cellular immunotherapies. APC, antigen-presenting cell; MHC, major histocompatibility complex; TCR, T-cell receptor; HLA, human leukocyte antigen; GVHD, graft-versus-host disease; NKG2D, natural killer group 2 member D; MICA/B, MHC class I chain-related protein A and B.

T cells illustrate how the immune system achieves a balance between precise recognition and rapid response. They originate in the bone marrow and mature in the thymus, where they are shaped into distinct categories (**Figure 1**). The majority carry αβ T cell receptors (TCRs), fitting neatly into the CD4+ helper or CD8+ cytotoxic roles, both tied to MHC restriction (23–25). Their dominance in lymphoid organs reinforces a predictable logic: recognition depends on antigen display through MHC. Yet a smaller group-γδ T cells-defies this structure. They act more like intuitive responders, less dependent on complex signaling. With rapid, MHC-independent responses and strong tissue localization, they occupy a hybrid space between innate and adaptive immunity (26–28). Their very unpredictability makes them attractive candidates for immunotherapy.

### Structural and functional distinctions between γδ and αβ T cells

The contrast between γδ and αβ T cells reflects two different modes of immune cognition. γδ TCRs, built from gamma and delta chains, recognize stress ligands, phosphoantigens, and non-peptide targets without requiring MHC mediation (29–31). αβ T cells, in contrast, rely on a slower, two-step verification: antigen presentation plus co-stimulatory signals (12, 32, 33). This design ensures accuracy but costs time. γδ T cells, needing only a single trigger such as phosphoantigen binding, respond with speed. Their roles-cytotoxic attack, cytokine release, tissue repair-emerge most vividly in epithelial and mucosal sites (34–36).

Clinically, they can attack tumors or pathogens that escape αβ surveillance through HLA downregulation (37–39). Their fast responses carve out a unique niche-promising in cancers where conventional αβ T cells fail because the tumor has learned to hide.

### Functional subsets of γδ T cells and disease correlation

**Figure 2:**
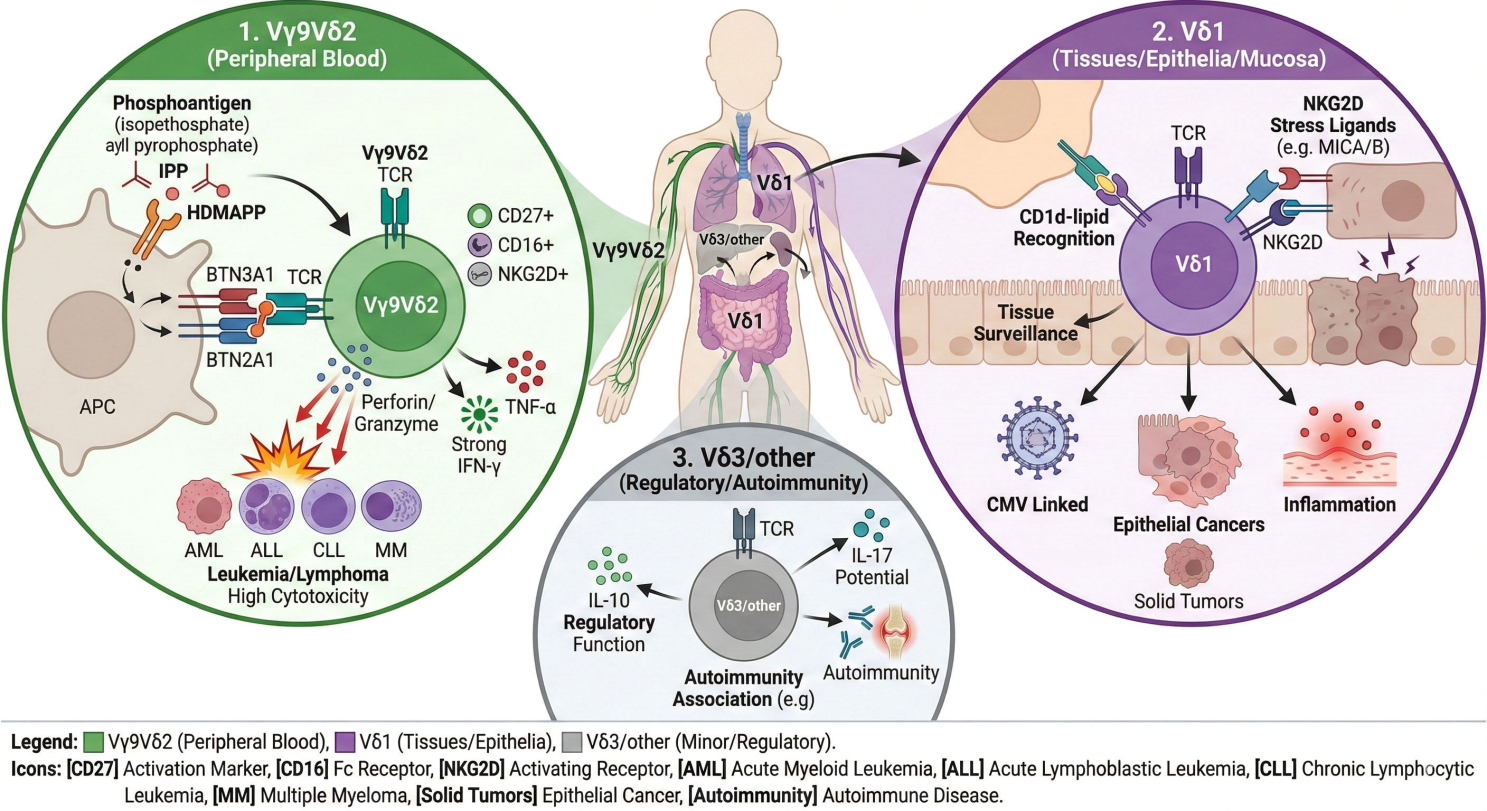
Functional subsets of γδ T cells and their disease relevance. γδ T cells are stratified into distinct subpopulations based on their δ-chain usage, each exhibiting unique tissue tropism and antigen recognition modalities (1) The Vγ9Vδ2 subset predominantly circulates in the peripheral blood. These cells recognize non-peptidic phosphoantigens (pAgs) accumulated in transformed or infected cells, a process critically mediated by the butyrophilin complex (BTN2A1/BTN3A1). Upon activation, they exert potent anti-tumor cytotoxicity against hematologic malignancies via perforin/granzyme degranulation and the secretion of pro-inflammatory cytokines (IFN-γ, TNF-α). (2) Conversely, the Vδ1 subset exhibits strong tissue tropism, primarily residing in mucosal barriers, skin, and the liver. They maintain epithelial surveillance by recognizing stress-induced ligands (e.g., MICA/B) via innate receptors such as NKG2D, as well as lipid antigens presented by CD1d. Their effector profiles are highly plastic, executing either potent cytotoxicity against epithelial tumors and cytomegalovirus (CMV)-infected cells, or exerting immunosuppressive functions via TGF-β and IL-10 secretion. (3) Other minor subsets, such as Vδ3 cells, are enriched in the liver and gut, where they recognize CD1d-presented lipids or Annexin A2. They primarily drive immunoregulatory networks by producing IL-4 and IL-10, playing essential roles in maintaining immune tolerance and modulating autoimmune responses. pAgs, phosphoantigens; BTN2A1/BTN3A1, butyrophilin subfamily 2 member A1/subfamily 3 member A1; IFN-γ, interferon-gamma; TNF-α, tumor necrosis factor-alpha; MICA/B, MHC class I chain-related protein A and B; NKG2D, natural killer group 2 member D; CD, cluster of differentiation; CMV, cytomegalovirus; TGF-β, transforming growth factor-beta; IL, interleukin.

The map of γδ T cell subsets reveals more than biological taxonomy (**Figure 2**); it marks possible roads for precision therapy. Vγ9Vδ2 cells, long adapted to recognize metabolic irregularities, align with the needs of anti-leukemic strategies, while Vδ1 cells, guardians of epithelial barriers, hold promise against solid tumors (**Table 1**).

**Table 1:** Characteristics and disease associations of γδ T cell subsets.

Subset	Key receptors/markers	Localization	Cytokine profile & functional plasticity	Associated diseases/roles
Vγ9Vδ2	TCR Vγ9Vδ2, CD27,CD45RA, NKG2D, BTN3A1	Peripheral blood, lymph nodes	Anti-tumor/Pro-inflammatory: IFN-γ, TNF-α (Th1-like). Regulatory: Can be skewed to IL-10 producing state by TME.	Malignancies: Cytotoxicity against AML, ALL, MM via phosphoantigen recognition. Infections: Expansion during viral (CMV, EBV) hematologic injury.
Vδ1	TCR Vδ1, CD1d, NKG2D, NKp30	Mucosal barriers, spleen, liver, bone marrow	Tissue surveillance: IL-17, TGF-β, IL-10. Dual role: Can exhibit potent cytotoxicity (Granzyme/Perforin) or profound immunosuppression.	Malignancies: Elevated in CLL and DLBCL (often exhausted/regulatory). Potent CAR-Vδ1 targets for relapsed AML. Autoimmunity: Dysregulated in ITP and AIHA.
Non-Vγ9Vδ2/Vδ1 (e.g., Vδ3)	TCR Vδ3, CD56, HLA-DR	Liver, gut, rare in blood	Regulatory: IL-10, IL-4, TGF-β. Associated with chronic inflammation and tolerance.	Post-HSCT: Implicated in immune reconstitution and establishing tolerance post-transplant; less defined in direct cytolysis.

TCR, T-cell receptor; IFN-γ, interferon-gamma; TNF-α, tumor necrosis factor-alpha; TGF-β, transforming growth factor-beta; IL, interleukin; AML, acute myeloid leukemia; ALL, acute lymphoblastic leukemia; MM, multiple myeloma; CLL, chronic lymphocytic leukemia; DLBCL, diffuse large B-cell lymphoma; CMV, cytomegalovirus; EBV, Epstein-Barr virus; IBD, inflammatory bowel disease; ITP, immune thrombocytopenia; AIHA, autoimmune hemolytic anemia.

### Translational implications: γδ T cells as universal therapeutic candidates

γδ T cells have several biological features that make them attractive candidates for cellular immunotherapy, including MHC-independent recognition, low alloreactivity, tissue-homing capacity, and rapid effector function. Their instinctive tissue-homing and rapid recognition capabilities make them natural candidates for therapies (40–42). Unlike αβ T cells, they can operate effectively within hypoxic and immune-suppressive tumor microenvironments effectively (12, 42, 43). Even more striking is their adaptability in the laboratory: they can be multiplied, shaped, and armed with engineered receptors while retaining cytotoxic activity (44–46).

While γδ T cells offer unique biological advantages that have the potential to complement existing therapies, realizing their role as universal, ‘off-the-shelf’ treatments requires overcoming translational hurdles. Their theoretical safety and applicability must be further rigorously validated in larger-scale clinical cohorts.

## γδ T cells in hematologic disorders: unmet needs and translational opportunities

Hematologic diseases, whether benign or malignant, represent not only biological disorders but also social and historical challenges, their incidence rising in parallel with aging societies, industrial exposure, and widening healthcare inequality. Even those labeled “non-malignant” conceal severe risks: severe aplastic anemia carries a 1-year mortality of 30–40%, while autoimmune hemolytic anemia reaches 8–23%, with warm autoimmune hemolytic anemia patients tripling their thromboembolism risk (47–49). Malignancies such as acute myeloid leukemia (AML), myelodysplastic syndromes (MDS), and lymphomas impose an even grimmer reality-relapse is common, survival is poor, and the burden falls heaviest on the elderly or those with hostile genetics (50–52). The five-year survival of elderly AML stays below 20%, while relapsed AML allows only half a year of life (53–56). Access to curative transplantation, limited to <5% in low- and middle-income regions, exposes the structural injustice of our medical systems (9, 57, 58). To this biology we must add the weight of society: exclusion, depression, and financial toxicity, with households spending over half their income on survival, deepening the spiral of loss (59–61).

### Matrix of unmet needs in hematologic disorders

Modern therapies, though impressive, falter across several dimensions:

- Efficacy: 40% of severe aplastic anemia patients fail to respond to immunosuppressive therapy (62, 63); 20–30% of diffuse large B-cell lymphoma relapses follow R-CHOP (64–66).
- Toxicity: Bortezomib induces neuropathy in 35–40% of myeloma patients (67–69); ATG drives infection (70, 71); splenectomy triples thrombosis (72, 73).
- Accessibility: Merely 35% of nations provide free immunosuppressive therapy (74, 75); transplant-related mortality lingers at 15–25% (76, 77); hematology specialist density spans from 2.3/100,000 in the rich world to 0.04/100,000 in the poor (78).
- Immune Evasion: Tumor microenvironments erode T cell vigor (79, 80); HLA loss in AML and MDS transforms therapy into futility (81, 82).

These limitations highlight the need for alternative immune-based therapeutic strategies, and the urgent call for new immune strategies.

### Rationale for γδ T cell–based interventions

**Figure 3:**
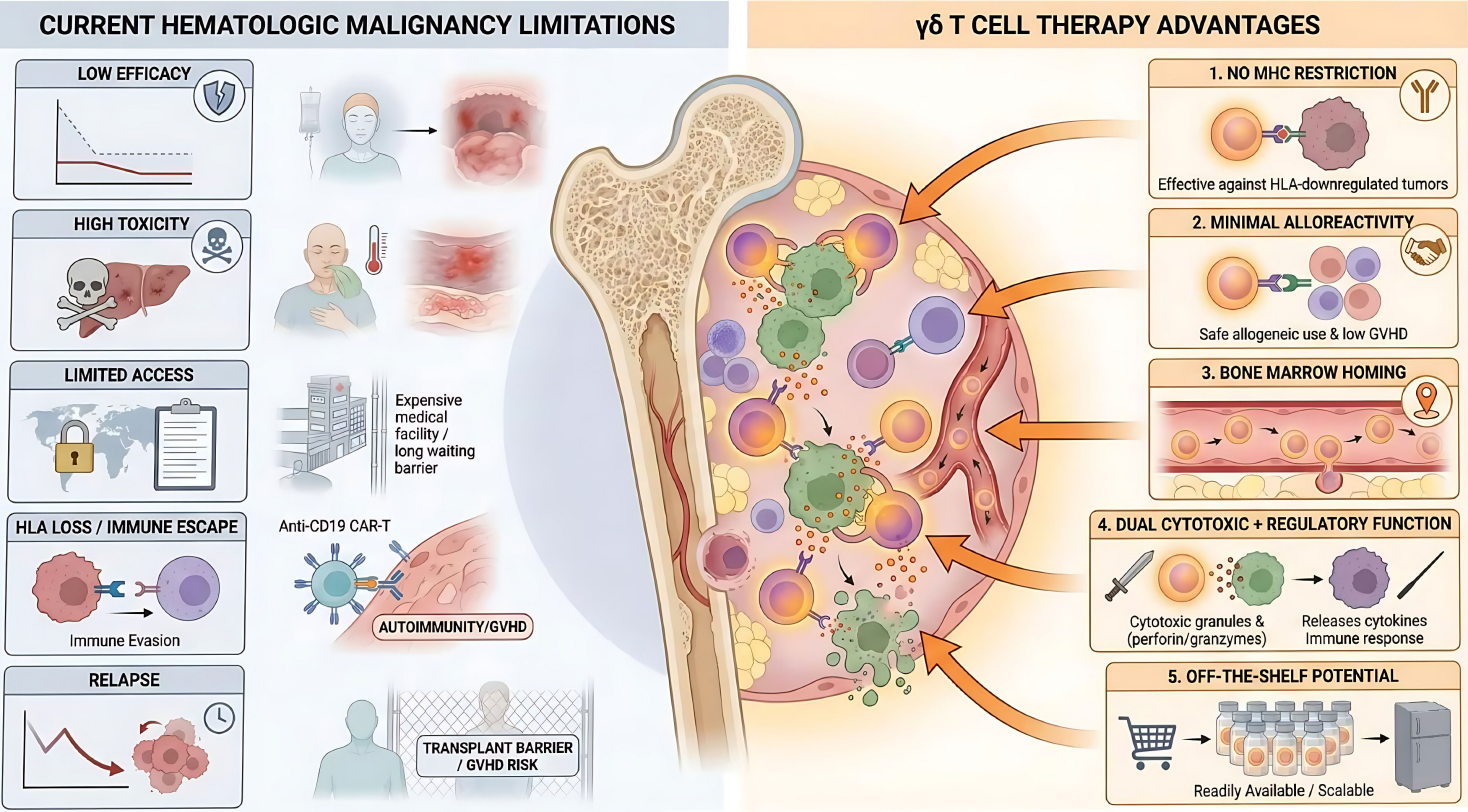
Translational advantages of γδ T cells in hematological diseases. Current conventional immunotherapies face critical clinical bottlenecks, including limited long-term efficacy driven by tumor immune evasion (e.g., HLA downregulation and antigen loss), severe safety toxicities such as cytokine release syndrome (CRS) and immune effector cell-associated neurotoxicity syndrome (ICANS), as well as restricted patient accessibility due to high manufacturing costs and prolonged vein-to-vein times. In contrast, γδ T cells offer intrinsic translational solutions to these barriers (1) Their MHC-independent recognition enables the direct targeting and eradication of HLA-deficient tumor variants that evade conventional αβ T cell surveillance. (2) Their unique biology confers a highly favorable safety profile, characterized by a minimal risk of graft-versus-host disease (GVHD) in allogeneic settings and a significantly lower incidence of severe CRS or ICANS. (3) Intrinsic chemokine receptor expression facilitates robust tissue homing and deep infiltration into protective tumor niches, such as the bone marrow. (4) Finally, their dual functionality allows them to execute direct potent cytotoxicity against malignant blasts while simultaneously orchestrating broader adaptive immune responses within the immunosuppressive tumor microenvironment (TME). HLA, human leukocyte antigen; CRS, cytokine release syndrome; ICANS, immune effector cell-associated neurotoxicity syndrome; MHC, major histocompatibility complex; GVHD, graft-versus-host disease; TME, tumor microenvironment.

Here, γδ T cells enter as evolutionary outliers with translational promise. Their ability to recognize stress antigens enables direct confrontation with HLA-deficient tumors such as relapsed AML or high-risk MDS (38, 83, 84). Unlike their αβ cousins, they can enter the marrow, sense cellular stress, and they may exert both cytotoxic and immunoregulatory functions, supporting their translational relevance. (**Figure 3**).

### Summary

Taken together, the biological properties of γδ T cells suggest clear translational relevance in hematologic diseases. Their MHC-independent recognition, functional plasticity, and low alloreactive potential make them promising candidates for addressing current therapeutic limitations. These features provide the rationale for further exploring their mechanisms of action and clinical applications.

## Mechanisms of γδ T cell activation and function

### Multimodal activation pathways enable broad-spectrum immune engagement

**Figure 4:**
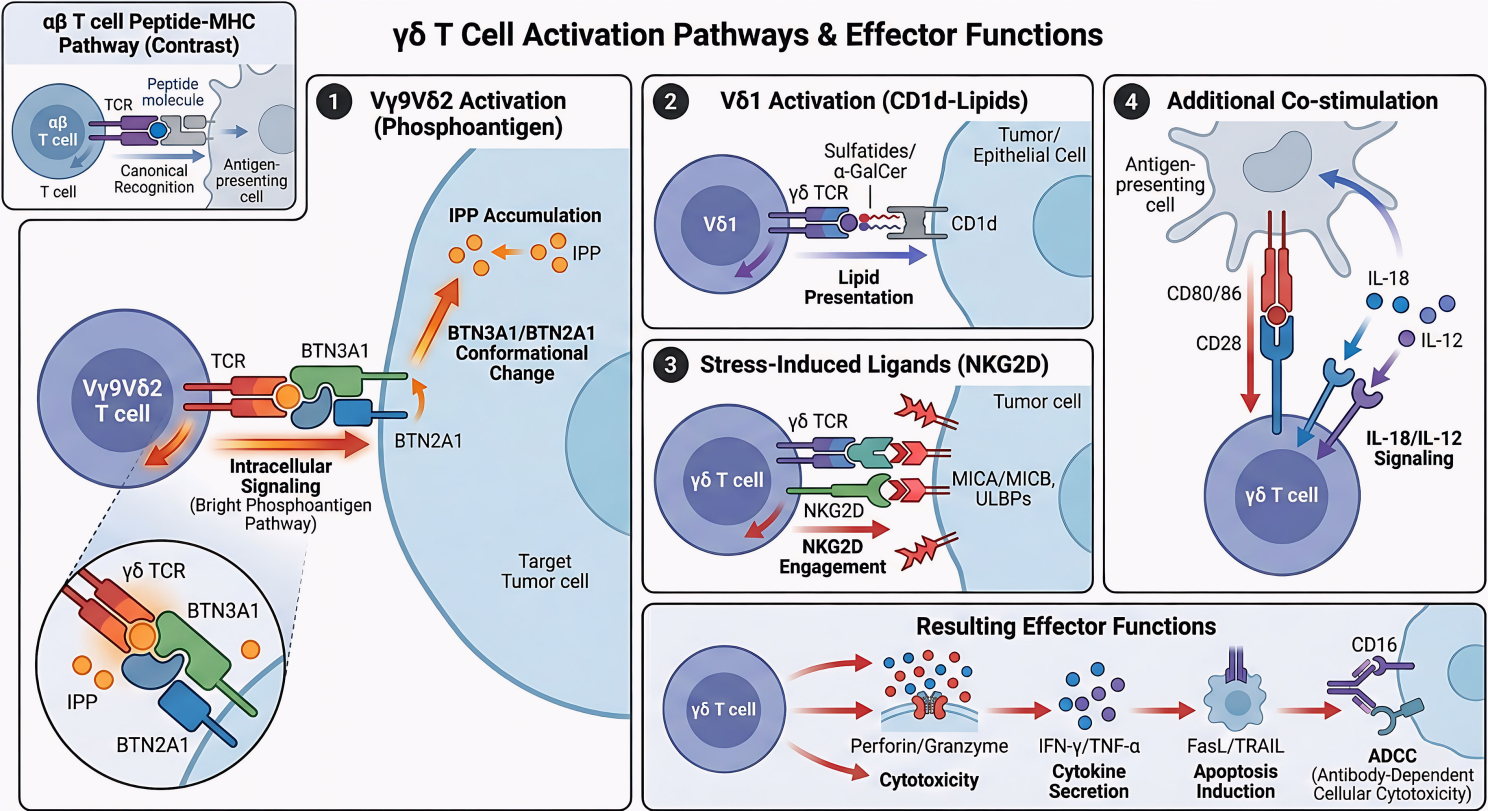
Multimodal activation mechanisms of γδ T cells. Conventional αβ T cells strictly depend on the recognition of specific peptide antigens presented by major histocompatibility complex (MHC) molecules on antigen-presenting cells (APCs). Conversely, γδ T cells utilize a broad, MHC-independent, and multimodal recognition system to detect transformed or infected cells. (1) Phosphoantigen Recognition: The γδ T-cell receptor (TCR) senses intracellular metabolic dysregulation by detecting accumulated phosphoantigens (pAgs, such as IPP or microbial HMBPP), a structural engagement mediated by the butyrophilin complex (BTN2A1 and BTN3A1). (2) Lipid Recognition: The γδ TCR independently recognizes lipid and glycolipid antigens presented by the non-classical MHC-like molecule CD1d. (3) Innate-like Stress Recognition: Synergizing with TCR signals, innate activating receptors such as NKG2D directly engage stress-induced surface ligands (e.g., MICA and MICB) that are upregulated on malignant cells. These converging activation pathways culminate in robust downstream effector functions, driving direct tumor cytotoxicity (via perforin and granzyme B degranulation) and the secretion of pro-inflammatory cytokines (IFN-γ and TNF-α) to orchestrate broader immune responses. APC, antigen-presenting cell; MHC, major histocompatibility complex; TCR, T-cell receptor; pAgs, phosphoantigens; IPP, isopentenyl pyrophosphate; HMBPP, (E)-4-hydroxy-3-methyl-but-2-enyl pyrophosphate; BTN2A1/3A1, butyrophilin subfamily 2 member A1/subfamily 3 member A1; CD1d, cluster of differentiation 1d; NKG2D, natural killer group 2 member D; MICA/B, MHC class I chain-related protein A and B; IFN-γ, interferon-gamma; TNF-α, tumor necrosis factor-alpha.

Unlike αβ T cells, γδ T cells are wide-spectrum detectors, able to detect non-peptidic cues across diverse metabolic and cellular stress pathways (85, 86). (**Figure 4**). Vγ9Vδ2 T cells in blood respond to phosphoantigens such as isopentenyl pyrophosphate, molecules that pile up in tumors where the mevalonate pathway falters (87–89). These phosphoantigens twist BTN3A1 and BTN2A1 into new conformations, unlocking recognition by γδ TCRs (90–92). Meanwhile, Vδ1+ γδ T cells register lipid antigens displayed by CD1d-sphingolipids, α-galactosylceramides-eliciting cytokines and repairing barrier tissues (19, 93). Other alarms arise from stress-induced ligands, MICA, ULBPs, HSP70, activating NKG2D signaling cascades, primarily in Vδ1+ cells, which trigger cytotoxic cascades and inflammatory waves in epithelial sites (94–96).

This multi-channel detection system allows γδ T cells to recognize cancers, infections, and damaged tissue without HLA restriction. Drugs like nitrogen bisphosphonates or CD1d agonists can tune these activation channels, selectively expanding tumor-reactive γδ T cells in controlled fashion, providing scalable therapeutic levers (97–99).

### Cytokine plasticity as a determinant of functional programming and disease stratification

γδ T cells exhibit high functional plasticity, which is heavily modulated by subset identity and the surrounding field. Vγ9Vδ2 cells usually polarize into Th1-like states, secreting IFN-γ and TNF-α via NF-κB and MAPK, amplifying tumor immunity and macrophage action (100–102). Yet in tumor microenvironments steeped in TGF-β and IL-6, γδ T cells may undergo phenotypic skewing into IL-10 or IL-4 producers, suppressing localized immune responses (103–105). Vγ6Vδ1 subsets in mucosa can be prolific IL-17A/F sources-bolstering barrier defense or, conversely, fueling chronic inflammation in autoimmunity and myeloid cancers (106–108).

Decoding this cytokine plasticity allows precision use of γδ T cells. IFN-γ–dominant subsets suit tumor therapy (109–111), while IL-10–producing γδ Tregs may drive resistance or be harnessed against inflammation (112, 113). Tuning these cytokine circuits offers both stratification and engineering routes for therapy.

### Dual cytotoxic and immune reprogramming functions drive antitumor efficacy

γδ T cells can precisely target lesions and also modulate the local microenvironment post-cytolysis. They are involved in mechanisms such as perforin/granzyme granules, FasL/TRAIL apoptosis, antibody-dependent cell-mediated cytotoxicity (ADCC), effectively eliminating malignant or infected cells (114, 115). Vγ9Vδ2 cells sense metabolic distress (116, 117), while Vδ1 cells engage stress ligands in tissues (118, 119). Beyond killing, γδ T cells rewire the tumor microenvironment: releasing chemokines such as CCL3, CXCL8, and TH1 cytokines that call in dendritic cells, NK cells, CD8+ T cells, creating long-term immune reinforcement (120, 121).

The dual characteristics of these cytotoxic effectors and immunomodulators make γδ T cells prime for immunotherapy. Adoptive transfers, including CAR-γδ T cells, exploit this versatility; early-phase human clinical trials and preclinical *in vivo* animal models show tumor suppression through both direct cytolysis and microenvironmental immune reprogramming (122–124). Pairing γδ therapy with checkpoint inhibitors or metabolic adjuvants may unlock resistant disease.

### Cooperative crosstalk with αβ T cells, NK cells, and dendritic cells underpins combination therapy

γδ T cells function as the connectors, bridging immune constellations. Through IFN-γ and GM-CSF they energize dendritic cells to sharpen antigen presentation (125–127); through 4-1BBL and chemokines they elevate NK cytotoxicity (128, 129); through chemotactic cues they draw in CD8+ T cells (130–132). Under inflammation, these synergies amplify, and γδ T cells also present antigens themselves or secrete AREG to mend tissues (133, 134).

Harnessing γδ T cells as adjuvants can amplify other therapies-αβ T cells, oncolytic viruses, dendritic cell vaccines. Rational co-culture systems and multi-cell adoptive designs are the emerging blueprints for such synergy.

### Functional plasticity as the basis for γδ T cell versatility in clinical applications

The versatility of γδ T cells lies in their ability to detect varied antigens, secrete a broad cytokine palette, combine cytotoxic with regulatory roles, and weave networks with other immune subsets (135–137). Their independence from MHC, their sensitivity to metabolism, and their effector-regulatory duality situate them at the crossroads of cancer, infection, and immune imbalance (138–140).

Functional plasticity is the key factor of γδ T cell therapy. Engineering expansion and modulation to favor IFN-γ+ cytotoxic or IL-10+ regulatory states offers precise tailoring (141–143). As clinical trials unfold, predictive biomarkers and refined manufacturing pipelines will determine how far this versatile immune axis can be pushed into practice.

## γδ T cells in hematologic malignancies: translational opportunities and challenges

### Critical limitations: clinical discrepancies, durability, and expansion variability

Even with remarkable therapeutic advances, hematologic malignancies-acute leukemia, lymphoma, and multiple myeloma (MM)-remain formidable. They are governed by invisible forces of relapse, resistance, and immune escape (144–146). In relapsed or refractory acute lymphoblastic leukemia (ALL), elderly patients face a five-year event-free survival below 30% (147–149). Acute myeloid leukemia (AML) offers no reprieve: in older cohorts, fewer than one in five reach the five-year mark, and relapse shortens life to 6–8 months (150, 151). Chronic lymphocytic leukemia (CLL), when burdened with TP53 mutations, yields less than 30% five-year survival, with resistance to BTK inhibitors surpassing 60% (152–154). In MM, patients carrying del(17p), t (4, 14), or TP53 mutations confront swift relapse (155). Lymphomas, from DLBCL to Hodgkin’s disease, present persistent clinical challenges, including immune evasion, CAR-T resistance, recurrence (156–158). Transplant relapse and minimal residual disease (MRD) negatively impact long-term patient survival and treatment durability, underscoring the need for innovative γδ T-cell immunotherapies (159–161).

### Mechanisms of action: subset-specific immune surveillance

γδ T cells connect innate and adaptive immunity to execute subset-specific surveillance. Vγ9Vδ2 cells perceive phosphoantigens such as IPP, deploying NKG2D to sense MICA/B, and strike with IFN-γ and granzyme B (162–164). Vδ1 cells, guardians of hidden niches, target stress ligands like CD123, infiltrating marrow sanctuaries and eradicating leukemic stem cells (LSCs) (165–167). In preclinical *in vitro* and murine xenograft models of AML and ALL, they have been shown to dismantle CD34+CD19+ reservoirs of relapse (168–170). Yet in CLL and MM, Vδ1 can be bent toward suppression, secreting IL-10 and IL-13 (171–173). Meanwhile, Vγ9Vδ2 cells are frequently inhibited by metabolically restrictive and checkpoint-upregulated microenvironments, PD-1, TIM-3, and mitochondrial collapse impairing their cytotoxic efficacy (174–176). These subset-specific dynamics mirror species diversity, and guide the design of targeted therapies.

### Clinical and preclinical evidence supporting γδ T therapies

#### Distinguishing preclinical efficacy from early-phase clinical outcomes

Preclinical models evaluating γδ T-cell therapies have consistently demonstrated anti-leukemic activity; however, translating these preclinical milestones into robust and predictable clinical outcomes remains a challenge. It requires a rigorous distinction between preclinical models and human clinical trials (177). In early-phase human clinical trials for ALL, expanded Vγ9Vδ2 reinfusion has achieved complete remission (CR) in approximately 38% of small patient cohorts, while early clinical evaluations of CD19-CAR-γδ T cells report response rates up to 67% (178–180). In human cohorts treated with decitabine combinations, the overall response rate (ORR) rose to 45% (181).

Conversely, strictly within preclinical murine xenograft models, experimental therapies such as NKG2D-CAR-γδ T cells and anti-CD123 CAR-Vδ1 cells have yielded profound leukemia clearance rates of 52% and 63% in AML, respectively. Similarly, in preclinical *in vivo* models of high-risk CLL, ROR1-CAR-Vδ1 and PD-1-CAR-γδ combinations have demonstrated potent tumor suppression (CR equivalent to 65% and ORR to 54% in murine cohorts), awaiting human translation (182–184).

Returning to human clinical data, Phase I trials involving patients with multiple myeloma (MM) infused with anti-BCMA CAR-Vγ9Vδ2 have achieved encouraging results, with a CR of 65% and an 82% overall survival (OS) rate at 24 months in small cohorts (185–187). In human Phase I dose-escalation trials for relapsed/refractory B-cell lymphoma, the allogeneic CD20-targeted CAR-Vδ1 therapy (ADI-001) has delivered an ORR of 62% and a CR of 41% without severe toxicities (188–190). Finally, retrospective correlative observations in human transplant recipients show that robust Vδ1/Vδ2 recovery significantly correlates with MRD negativity (91%) and a reduction in relapse risk (HR = 0.32) (189, 191–193).

Current human clinical data are predominantly restricted to Phase I/II safety and dose-escalation trials with small patient cohorts (typically N < 20). These early-phase clinical observations confirm the exceptional safety profile of allogeneic γδ T cell infusions (with negligible severe GVHD), but they also reveal that the sustained clinical efficacy and *in vivo* persistence often fall short of the dramatic responses observed in animal models. To provide clear delineation, the following sections will explicitly categorize the evidence levels, and a comprehensive summary of recent patient-derived clinical trial data is synthesized in **Table 2**.

**Table 2:** Recent key clinical trials of γδ T cell therapies in hematologic malignancies.

Disease	Intervention/subset	Clinical trial phase	Cohort/N	Main outcomes & efficacy	Safety profile/adverse events	Ref
Relapsed/Refractory B-NHL	LUCAR-G39D (Allogeneic Anti-CD20/CD19 Dual-CAR γδ T cells)	Phase I (NCT06395870)	N=12	88% of evaluable patients showed decreased ctDNA; encouraging early ORR and durability.	Manageable safety. Infections observed (mostly Grade 1/2), no severe ICANS.	*Blood* (2025) 146:552698
High-Risk AML (Post-alloSCT)	Ex vivo expanded donor-derived allogeneic Vγ9Vδ2 T cells	Phase I (NCT05015426)	N=12	17% relapse rate at 11 months median follow-up. Majority remained in CR.	Highly favorable. No dose-limiting toxicities or severe acute GVHD induced.	*VJHemOnc* (2025); *Front Immunol*
B Cell Malignancies (MCL focus)	ADI-001 (Allogeneic CD20-CAR Vδ1 T cells)	Phase I (NCT04735471)	N=10 (MCL cohort)	ORR 80%, CR 60%; mDoR 17.5 months. Highly competitive with approved agents.	No Grade ≥3 CRS or ICANS. No GVHD reported.	*ASCO*/Adicet Data (2024-2025)
CD123+ R/R AML & MDS	LAVA-1266 (CD123-targeting bispecific Vγ9Vδ2-T cell engager)	Phase I (ACTRN12624001214527)	Escalation cohort (~50 planned)	Dose escalation ongoing. Preclinical validation showed potent CD123+ blast clearance.	Interim safety shows limited off-target toxicity and manageable cytokine profiles.	*WSLHD Repository* (2025)

R/R, relapsed/refractory; B-NHL, B-cell non-Hodgkin lymphoma; alloSCT, allogeneic stem cell transplantation; MCL, mantle cell lymphoma; MDS, myelodysplastic syndromes; ORR, overall response rate; CR, complete remission; PR, partial response; mDoR, median duration of response; ctDNA, circulating tumor DNA; CRS, cytokine release syndrome; ICANS, immune effector cell-associated neurotoxicity syndrome; GVHD, graft-versus-host disease.

### Epigenetic and transcriptional regulation of γδ T cells by the hematologic tumor microenvironment

The hematologic tumor microenvironment (TME) exerts profound modulatory effects on γδ T cell polarization, often driving an immunosuppressive phenotype through complex epigenetic and transcriptional reprogramming. Recent advances, including spatial transcriptomic analyses, have unveiled highly compartmentalized niches that dictate immune evasion (194).

A prominent example is observed within the diffuse large B-cell lymphoma (DLBCL) microenvironment. Spatial mapping reveals that tumor-derived signals heavily influence the local γδ T cell state (195). Transcriptional networks driven by the TGF-β pathway—specifically through the TGFB1-IL2RA signaling axis—can skew γδ T cells toward an IL-10-producing, regulatory phenotype (γδ Tregs), effectively suppressing localized cytotoxic responses and promoting lymphoma progression.

Furthermore, epigenetic remodeling within the hematologic TME upregulates inhibitory checkpoints. The LGALS9-CD44 axis has recently emerged as a critical immune checkpoint regulator in this context (196). Galectin-9 (LGALS9), secreted by malignant cells or associated stroma, engages CD44 on infiltrating γδ T cells. This interaction initiates downstream transcriptional programs that drive functional exhaustion, metabolic suppression, and premature apoptosis. Conversely, in acute myeloid leukemia (AML), the hypoxic bone marrow niche drives epigenetic silencing of NKG2D expression via histone deacetylase (HDAC) activity, further paralyzing Vδ1 subset surveillance (197–199). Understanding these specific spatial and transcriptional networks is crucial for reversing TME-induced exhaustion in γδ T cell therapies.

### Translational barriers and strategies

#### Critical limitations: clinical discrepancies, durability, and expansion variability

Metabolic suppression-IDO consuming tryptophan, arginase draining arginine-further darkens their energy (200, 201). Suppressive ligands like Galectin-9 and IL-13 further exacerbate localized immunosuppression (202, 203). Yet pathways to escape exist: checkpoint blockade (anti-PD-1, TIM-3, LAG-3), metabolic interventions (epacadostat, CB-839), and cytokine fuel (IL-2, IL-15) (204–206). Engineering expands their reach-dual CARs (CD19/CD20), IL-15R, bispecific engagers (207–209). Combinations-cytarabine with γδ T cells (AML, overall response rate: 58%), pomalidomide with Vδ1 inhibition (MM, overall response rate: 45%), CD19/CD20 CARs in diffuse large B-cell lymphoma (relapse to 15%)-illuminate synergistic effects (210–212). Graft engineering, purging αβ TCRs, lessens GVHD while preserving GVL, bending the arc of immunity (213–215).

#### Rational engineering and CAR design considerations for γδ T cells

To overcome the hostile hematologic TME, rational engineering of Chimeric Antigen Receptors (CARs) specific to γδ T cells is imperative. Unlike conventional αβ T cells, γδ T cells benefit from unique co-stimulatory requirements (216).

Optimized Co-stimulatory Domains: Standard CD28 or 4-1BB endodomains may not fully capitalize on γδ T cell biology. Incorporating NKG2D or CD27 intracellular signaling domains into the CAR construct has been shown to synergize with innate TCR signaling, reducing activation-induced cell death (AICD) and enhancing long-term persistence (217).

“Armored” CAR Constructs: To combat transcriptional exhaustion *in vivo*, engineering γδ T cells to constitutively express supportive cytokines (e.g., tethered IL-15 or IL-18) maintains metabolic fitness without the need for systemic cytokine administration (218).

Logic-Gated Systems: To mitigate on-target, off-tumor toxicity in myeloid malignancies (such as targeting CD123 or CLL-1), AND-gated or OR-gated CAR systems are being evaluated. These require the simultaneous engagement of a synthetic tumor antigen and an innate stress ligand (via native NKG2D), ensuring highly localized cytolysis exclusively within the leukemic marrow niche (219).

### Research recommendations and priority areas

There are five priority areas as follows:

- Stratify patients by Vδ1/Vδ2 balance and checkpoint expression;
- Improve *in vitro* expansion with zoledronic acid or IL-15;
- Create disease-specific CAR-γδ constructs-CD123 for AML, ROR1 for CLL, BCMA for MM;
- Pair metabolic reprogramming with checkpoint inhibition;
- Advance phase II/III trials in minimal residual disease-positive and transplant-relapsed cohorts.

The evidence level is strongest in AML, ALL, and MM-marked “High” priority. CLL and lymphomas remain less charted, yet with engineering and microenvironmental mastery, they too may yield.

## γδ T cells in hematologic disorders beyond malignancies

### Immune tolerance reconstruction in autoimmune hematologic disorders (AIHA, ITP, AA)

Autoimmune hematologic disorders, such as autoimmune hemolytic anemia, immune thrombocytopenia, and aplastic anemia, are the outcome of immune system attacking autologous blood cells. Past methods-glucocorticoids, rituximab, immunosuppressants-seek to suppress, but not to solve (220–222). They transiently suppress inflammation but rarely restore long-term immune tolerance. γδ T cells, with their unique and flexible role, step forward as modulators of dysregulated immune responses. In hemolytic anemia, experiments in mice show that activated γδ T cells drive B-1 cells, which in turn exacerbate autoantibody-mediated destruction (223–225). But under clear conditions, the same γδ T cells, secreting IL-10 and TGF-β, can suppress autoreactive B cell clones, and keep immune tolerance (226–228). In ITP, γδ T cells bear two faces: some hasten platelet loss, while others strengthen thrombopoietin receptor signals, aiding Tregs in restraining autoimmunity (229–231). In aplastic anemia, they may stand guard over stem cells, or they may strike against them, depending on the camp they take (232–234). Thus in the field of immunotherapy, a viable strategy has been identified: to select the appropriate subset of immune cells, design suitable stimuli to activate these cells, enrich the Vδ1+ cells that produce IL-10, and place them at the forefront of immune reconstruction. This process points to a potential approach that could achieve long-term disease remission and prevent recurrence.

### γδ T cells as adjunctive agents in infection-induced hematologic injury

Factors that activate the immune system are not limited to autoimmune diseases; sometimes they appear in the form of infections—such as HIV, tuberculosis, and malaria—which attack the blood and bone marrow, disrupting the human immune system (235–237). Conventional therapy clears microbes, but cannot support the restoration of the hematopoietic niche (238–240). Here again, γδ T cells have their duty. In tuberculosis, Vγ9Vδ2 T cells sense phosphoantigens, call BTN3A1 into play, and release IFN-γ and granulysin, killing the invader (241–243). In HIV infection, the subset distribution shifts: the Vδ2+ population contracts while the Vδ1+ population expands, disrupting the CD4+/CD8+ balance (244–246). Yet in those exposed but uninfected, γδ T cells secrete chemokines that resist the virus-proof of their protective power (247, 248). In malaria, γδ T cells rally Th1-type defenses and hold the line against parasitemia, though prolonged struggle wears them down (249–251). What is to be done? Restore their vigor, engineer their strength, precondition with cytokines, combine them with marrow-supporting agents-thus one may both strike the pathogen and preserve the niche. This is a new road in translational strategy.

### Subset optimization and preconditioning for functional specificity

γδ T cells are not a single entity but are diverse. Some cells secrete IFN-γ and TNF-α to activate immune cells, while others produce IL-10 and TGF-β to suppress immune activation (252–254). Vγ9Vδ2 cells are renowned for cytotoxicity and defense against infection (255–257). Vδ1+ cells, with tissue-homing and tolerogenic gifts, play another part (258). Preclinical work shows: by pre-treating immune cells with specific cytokines (IL-2, IL-15, IL-21), their effector capabilities can be enhanced or strengthened (259–261). On the other hand, when these cells are exposed to inhibitory cytokines such as IL-10 or TGF-β, they shift towards a more suppressive or regulatory role, which helps maintain the balance of the immune system, prevent overreaction, and reduce inflammation (18, 262, 263). Sorting by CD27, CD45RA, and shaping with epigenetic tools brings fidelity and order. For the clinic, precision must match condition: Vδ2 cells rich in IFN-γ for clearing infection, Vδ1 cells rich in IL-10 for rebalancing autoimmunity (264–267). Thus, by tailoring expansion, we avoid waste, reduce danger, and secure maximum benefit in hematologic disease.

### Translational priorities and clinical development directions

Though evidence grows, γδ T cells are still rare in clinical trials of blood disease. This is a shortcoming, and must be corrected. Three priorities lie before us: first, use IL-10+ γδ T cells to induce tolerance in stubborn autoimmune disorders; second, deploy γδ T cell transfer as an ally against drug-resistant tuberculosis; third, build subset-specific platforms with safety switches, to command and recall the cells at will. Trials must not be blind-they must track subsets by biomarkers, profile them at single-cell level, and measure outcomes not only by response, but by reconstitution, relapse, and side effects. To make the therapy accessible, prepare off-the-shelf products from healthy donors, expand them feeder-free, and secure GMP standards. In conclusion, while γδ T cells represent a promising vector for cellular immunotherapy, they are currently in the developmental and optimization stage. Addressing the critical limitations of *in vivo* persistence, functional exhaustion, and manufacturing consistency is paramount for their future clinical integration.

## Therapeutic potential of targeting γδ T cells

### Overview of γδ T cell–based therapeutic platforms

**Figure 5:**
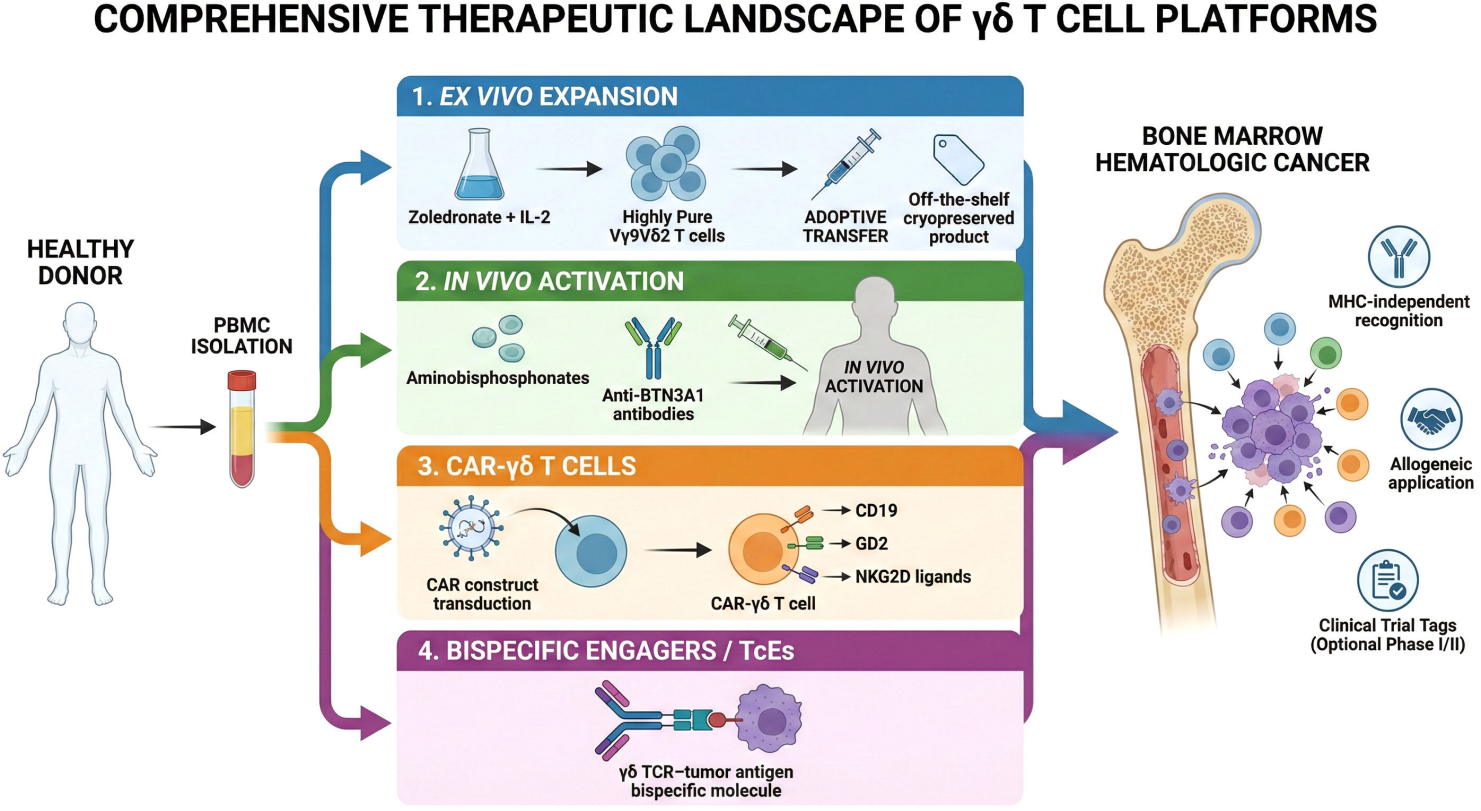
Therapeutic platforms harnessing γδ T cells for cancer immunotherapy. Current translational strategies to exploit the anti-tumor potential of γδ T cells are broadly categorized into four distinct modalities (1). *In vivo* Pharmacological Activation: Systemic administration of aminobisphosphonates (e.g., zoledronic acid) or synthetic phosphoantigens (pAgs) directly stimulates the expansion and activation of endogenous Vγ9Vδ2 T cells within the patient. (2) Adoptive Cell Transfer (ACT): Autologous or allogeneic γδ T cells (sourced from peripheral blood or umbilical cord blood) are isolated, massively expanded ex vivo using specific cytokines and pAgs, and subsequently reinfused into the patient to execute direct cytolysis. (3) Bispecific Antibodies (BsAbs)/Engagers: Engineered bispecific molecules physically crosslink the γδ T-cell receptor (TCR) to specific tumor-associated antigens (Ag), forcibly redirecting γδ T cell cytotoxicity toward malignant targets. (4) Chimeric Antigen Receptor (CAR) Engineering: γδ T cells are genetically modified to express CAR constructs, effectively synergizing their innate, MHC-independent tissue-homing capabilities with high-affinity, targeted tumor antigen recognition. ACT, adoptive cell transfer; BsAb, bispecific antibody; CAR, chimeric antigen receptor; pAgs, phosphoantigens; TCR, T-cell receptor; Ag, antigen; MHC, major histocompatibility complex.

The field of γδ T cell therapy has opened up many paths (117, 268, 269) (**Figure 5**). We can use adoptive cell transfer, we can build chimeric antigen receptors (CAR), we can design bispecific antibodies, and we can apply pharmacologic activators like bisphosphonates. We can also combine these approaches. The strength of γδ T cells across these platforms lies in their natural movement to tissues and their swift effector function (270–272) (**Table 3**). These are advantages that, if fully used, can make them important in cancer immunotherapy.

**Table 3:** γδ T cell therapeutic platform overview.

Platform	Example product/modality	Target	Indication	Clinical status
ACT	Expanded Vγ9Vδ2 T cells	–	AML, glioblastoma	Phase I–II
CAR-γδ T	ADI-001 (CD20-CAR-Vδ1), CD19 CAR-Vδ2	CD20, CD19	B-cell lymphoma	Phase I (Fast Track)
Bispecific γδ T engagers	BsAb (e.g., CD3xγδTCR)	Tumor Ag/γδ TCR	Solid tumors	Preclinical–Early Trial
Pharmacologic activators	Zoledronic acid + IL-2	FPPS/BTN3A1	Breast cancer, AML, post-HSCT	Pilot trials
Combinatorial	γδ T + ICI/radiotherapy/chemotherapy	–	Melanoma, lung cancer	Exploratory Phase

ACT, adoptive cell transfer; CAR, chimeric antigen receptor; BsAb, bispecific antibody; Ag, antigen; FPPS, farnesyl pyrophosphate synthase; BTN3A1, butyrophilin subfamily 3 member A1; ICI, immune checkpoint inhibitor; AML, acute myeloid leukemia; HSCT, hematopoietic stem cell transplantation.

### Translational bottlenecks and proposed solutions

**Figure 6:**
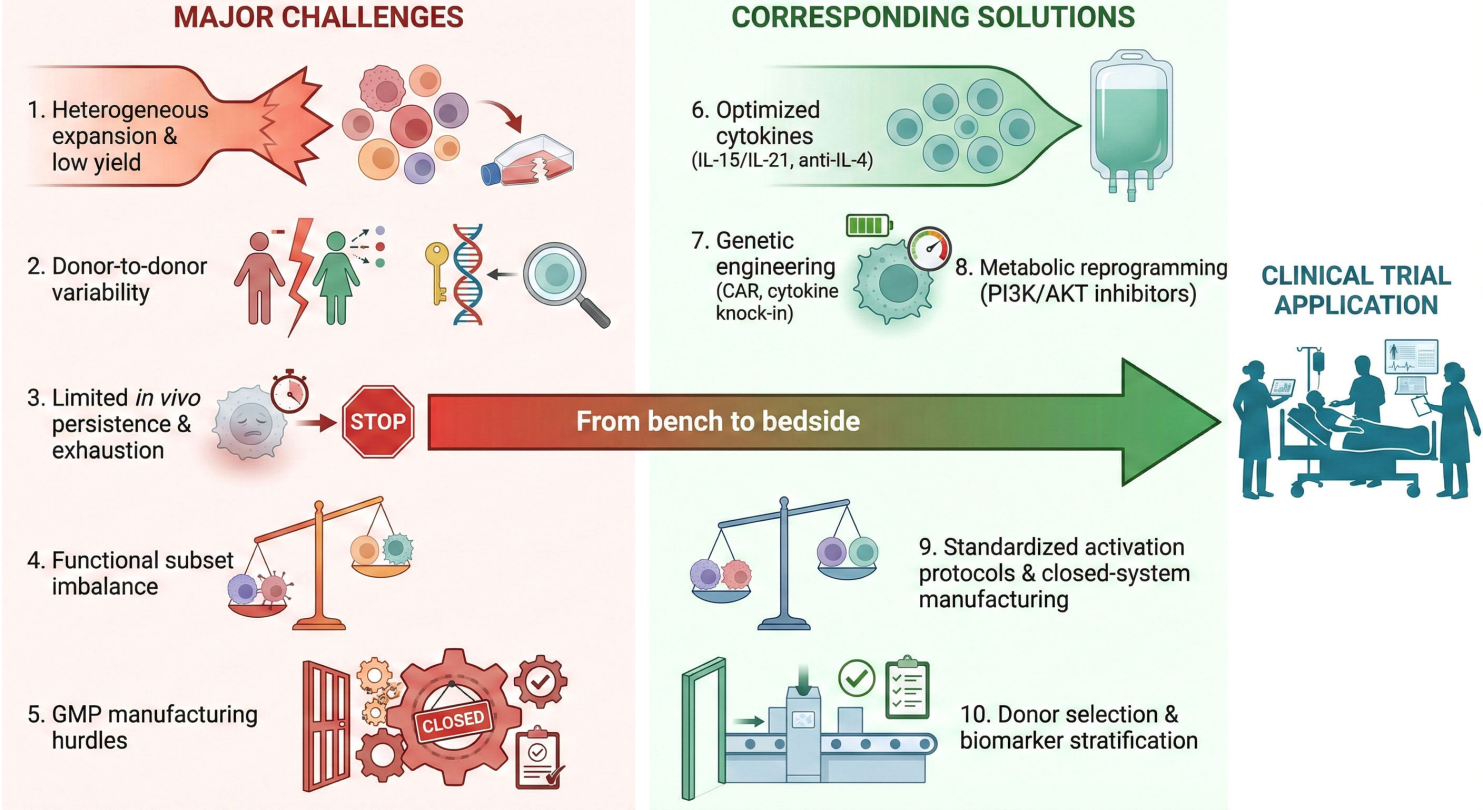
Translational bottlenecks and solutions for γδ T cell therapy. The successful transition of γδ T cell therapies from bench to bedside is currently hindered by several critical challenges. These include (1) heterogeneous ex vivo expansion resulting in low cell yields, (2) significant donor-to-donor intrinsic variability, (3) limited *in vivo* persistence driven by rapid functional exhaustion, (4) imbalances among functional subsets (e.g., cytotoxic versus regulatory phenotypes), and (5) stringent Good Manufacturing Practice (GMP) production hurdles. To overcome these barriers, corresponding multi-dimensional solutions are actively deployed: (6) utilizing optimized cytokine combinations (e.g., IL-15, IL-21, and anti-IL-4 antibodies) to selectively drive the proliferation of effector subsets; (7) employing genetic engineering (such as CAR constructs or cytokine knock-ins) to enhance autonomous survival; (8) applying metabolic reprogramming strategies (e.g., PI3K/AKT pathway inhibitors) to prevent terminal exhaustion; (9) establishing standardized activation protocols within closed-system manufacturing to ensure product consistency; and (10) implementing precise donor selection and biomarker stratification. The integration of these strategies is essential to optimize the efficacy, reproducibility, and scalability of clinical trial applications. GMP, Good Manufacturing Practice; IL, interleukin; CAR, chimeric antigen receptor; PI3K, phosphoinositide 3-kinase; AKT, protein kinase B.

Despite the compelling rationale for γδ T cell-based therapies, a critical appraisal of current literature reveals substantial limitations that must temper clinical expectations. Addressing these limitations will require improvements in expansion protocols, product consistency, and *in vivo* persistence. The answer lies in improving methods of expansion, reducing variability, and finding ways to strengthen persistence (**Figure 6**). Only then can we turn promise into real effect.

First, there is a marked discrepancy between preclinical success and clinical efficacy. While *in vitro* assays and murine xenograft models consistently show robust cytolytic clearance of hematologic blasts by expanded γδ T cells, these models fail to fully recapitulate the complex, immunosuppressive human tumor microenvironment (TME) (273). Human phase I trials frequently report modest overall response rates, highlighting that preclinical cytotoxicity does not seamlessly translate to *in vivo* tumor eradication, largely due to the human TME’s capacity to induce rapid anergy (274).

Second, the durability of clinical responses remains a significant limitation. Unlike CD19-targeted αβ CAR-T cells, which can exhibit prolonged *in vivo* expansion and memory formation, adoptively transferred γδ T cells often suffer from rapid clearance and a lack of long-term persistence without continuous exogenous cytokine support (e.g., IL-2 or IL-15) (275). *In vivo*, these cells rapidly upregulate exhaustion markers such as PD-1 and TIM-3 upon engaging the leukemic niche, drastically curtailing their sustained effector function and leading to transient responses.

Finally, manufacturing variability remains a profound translational bottleneck. The ex vivo expansion of γδ T cells (primarily the Vγ9Vδ2 subset) using aminobisphosphonates is heavily donor-dependent. High variability in final cell yield, purity, and functional fitness is frequently observed, particularly when utilizing autologous cells from heavily pre-treated patients whose baseline immune compartments are already compromised (276). This donor-to-donor heterogeneity severely challenges the development of standardized, reproducible ‘off-the-shelf’ allogeneic products, underscoring the necessity for optimized, feeder-free GMP manufacturing protocols before widespread clinical adoption can be realized.

## Challenges and future directions in γδ T cell therapy

### Challenges in γδ T cell product consistency

In the short period of two years, it will be necessary to set the standards of control and to define markers of function. Over five years, practice must converge toward protocols that are uniform and suitable for GMP facilities in many centers.

### Unresolved biology and targeting of γδ TCRs

The next question is biological in nature. Although experiments in the laboratory suggest potential, the full understanding of γδ T cells has not been attained. One observes that no single transcription factor defines a subtype, so knockout mice cannot be easily constructed. Vδ1 and Vγ9Vδ2 differ, but the precise criteria for choosing one or the other in patients remain uncertain (277). Their migration in tissues, their response to gradients of phosphoantigen, and their ability to enter tumors are all incompletely known. For progress, new tools are required: organoid models, single-cell technologies, and gene-engineered animals such as TCRγ-KO mice. The task of the coming five years is to map these interactions in a systematic way, and to obtain a coherent picture of the γδ TCR in relation to tumor ligands.

### Clinical standardization and biomarker gaps

Turning to the clinic, one again meets the problem of divergence. Each center defines subpopulations in its own manner; flow cytometry gates are not alike (278). Some investigators emphasize function, others emphasize purity. This makes results difficult to compare. Yet there are first indications of common measures: levels of HMBPP, receptors such as NKG2D or CD16A, and metabolic markers like SDHA (279–281). It follows that the near goal-within two years-is a shared immunophenotyping panel and infusion method. At five years, the requirement will be greater: biomarkers must guide which patients should receive therapy, and pharmacodynamic measures must enter into trial design. Only thus can results be both reproducible and useful.

### Strategic priorities and translational timeline

From these facts arises the need for order in research. The immediate task is practical: γδ T cells must be expanded with little exhaustion and tested for survival after infusion. In the next five years, more advanced products-CAR-modified γδ T cells and bispecific adaptors-should be tested in both solid and blood cancers. The longer view, of about eight years, is that one should aim for cells prepared in advance, edited for resistance to chemotherapy, and capable of durable tumor control. This vision depends on three further steps: reliable biomarkers to predict effect, careful study of dose, and means to overcome suppression by the tumor itself.

### Building collaborative platforms and data sharing

Finally, such work cannot be done by single groups in isolation. One must imagine a consortium of centers, in which patients are enrolled by uniform rules, cells are prepared by common methods, and data are analyzed with shared tools. Bioinformatics will integrate multi-omics results with clinical outcomes, and machine learning will help to find predictive signs (282–284). Within two years, agreements for sharing data and establishing registries must be put in place. In five years, one should expect multi-center trials, employing harmonized products and common endpoints. Only by such collaboration can the path to regulatory approval and practical treatment of patients be made smooth.

### Safety profiles and risk management in γδ T cell therapy

The unique safety profile and risk considerations of γδ T cells must be rigorously identified and managed.

First, Immune-Mediated Toxicities: Although severe Cytokine Release Syndrome (CRS) and Immune Effector Cell-Associated Neurotoxicity Syndrome (ICANS) are significantly less frequent compared to conventional autologous αβ CAR-T therapies, transient grade 1–2 CRS is frequently observed, particularly upon *in vivo* activation (e.g., using aminobisphosphonates) or following the infusion of highly active CAR-γδ T cells (285).

Second, Pathological Plasticity and Unintended Regulatory Effects: The extreme inherent plasticity of γδ T cells poses a theoretical risk of pathological transdifferentiation within the tumor microenvironment (TME). Under conditions of chronic inflammation or specific cytokine milieus (such as TGF-β rich niches), effector subsets may skew towards an unintended regulatory phenotype (γδ Tregs) or an IL-17-producing (γδT17) state. These subsets can inadvertently suppress localized anti-tumor immunity, promote angiogenesis, and potentially exacerbate co-existing autoimmune conditions (286).

Furthermore, manufacturing variability remains a critical risk factor for clinical translation. The intrinsic heterogeneity of donor starting material leads to inconsistent ex vivo expansion rates and variable product purity, directly impacting dose reproducibility. Establishing standardized quality control markers and robust, reproducible GMP protocols is a paramount priority for comprehensive risk mitigation (287).

## Conclusions and perspectives

### Integrated summary: the special position of γδ T cells in immunological strategy

γδ T cells stand apart from the conventional αβ lineage because they operate independently of MHC constraints and directly recognize stress or malignant signals (288–290). This independence grants them the quality of immediate response and the vigor of innate defense, while also bearing adaptive potential. Their distribution is not uniform: Vδ1+ γδ T cells guard the mucosa and epithelial outposts of the body, whereas circulating Vγ9Vδ2+ γδ T cells prove powerful against hematologic malignancies (118, 291, 292). In practice, clinical evidence has shown that in ALL and AML, infiltration by these cells correlates with improved survival (293–295).

### Translational vision: broadening application from laboratory insight

Antigens such as CD123 become vulnerable to γδ T cell targeting even when tumor HLA-I is lost (296–298). Clinical studies suggest that patients with post-transplant γδ T-cell recovery >10% may have improved survival outcomes after transplant attain superior survival-proof of their graft-versus-leukemia role (299–301). In alliance with chemotherapy, radiotherapy, or checkpoint blockade, their effect is magnified (302–304). Further, bispecific antibodies and lymphodepletion schemes may rouse their force in refractory AML or CLL (305–307). Beyond malignancy, they can aid repair in aplastic anemia, though under other conditions they may intensify autoimmunity, as in hemolytic anemia (307–310). Thus, to distinguish subsets with single-cell sequencing is the path to stratified and precise application (311–313).

### Strategic priorities and collaborative frameworks

To realize the clinical potential of γδ T cells, standardized expansion protocols and precise subset stratification methods must be established, with their efficacy validated through rigorous clinical trials (314–316). Robust biomarkers must be developed to guide patient stratification, and multicenter collaborations are essential to accelerate clinical translation. Scholars and clinicians should collaborate to bridge the gap between basic immunobiology and clinical applications, effectively overcoming key barriers such as functional exhaustion, persistent *in vivo* limitations, and production variability.

## Glossary

HLA: Human Leukocyte Antigen
MHC: Major Histocompatibility Complex
AML: Acute Myeloid Leukemia
ALL: Acute Lymphoblastic Leukemia
CLL: Chronic Lymphocytic Leukemia
MM: Multiple Myeloma
AIHA: Autoimmune Hemolytic Anemia
ITP: Immune thrombocytopenia
AA: Aplastic Anemia
CAR: Chimeric Antigen Receptor
SAA: Severe Aplastic Anemia
OS: Overall Survival
CD4: Cluster of Differentiation 4
CD8: Cluster of Differentiation 8
GMP: Good Manufacturing Practice
Vγ9V: T Cell Receptor Gamma 9 Delta 2 Subset
Vδ1: T Cell Receptor Delta 1 Subset
MDS: Myelodysplastic Syndromes
DLBCL: Diffuse Large B-Cell Lymphoma
CHOP: Cyclophosphamide, Doxorubicin, Vincristine, Prednisone
IDH: Isocitrate Dehydrogenase
ATG: Anti-Thymocyte Globulin
IST: Immunosuppressive Therapy
IPP: Isopentenyl Pyrophosphate
MICA: MHC Class I Chain-related Antigen A
HSP70: Heat Shock Protein 70
IFN-γ: Interferon Gamma
TNF-α: Tumor Necrosis Factor Alpha
NF-κB: Nuclear Factor Kappa-light-chain-enhancer of Activated B Cells
MAPK: Mitogen-Activated Protein Kinase
TGF: Transforming Growth Factor Beta
IL-6: Interleukin-6
IL-10: Interleukin-10
IL-4: Interleukin-4
Vγ6V: T Cell Receptor Gamma 6 Delta 1 Subset
IL: Interleukin
TRAIL: TNF-Related Apoptosis-Inducing Ligand
ADCC: Antibody-Dependent Cell-mediated Cytotoxicity
CCL3: C-C Motif Chemokine Ligand 3
CXCL8: C-X-C Motif Chemokine Ligand 8 (Interleukin-8)
TH1: T Helper 1 Cells
NK: Natural Killer Cell
GM-CSF: Granulocyte-Macrophage Colony-Stimulating Factor
AREG: Amphiregulin
TP53: Tumor Protein p53
BTK: Bruton Tyrosine Kinase
MRD: Minimal Residual Disease
CD123: Cluster of Differentiation 123
CD34: Cluster of Differentiation 34
CD19: Cluster of Differentiation 19
IL-13: Interleukin-13
PD-1: Programmed Cell Death Protein 1
TIM-3: T-cell Immunoglobulin and Mucin-domain containing-3
CR: Complete Remission
ORR: Overall Response Rate
Vδ2: T Cell Receptor Delta 2 Subset
BCMA: B-cell Maturation Antigen
ADI-001: CD20 Chimeric Antigen Receptor Vδ1 γδ
HR: Hazard Ratio
IDO: Indoleamine 2,3-Dioxygenase
LAG-3: Lymphocyte-activation gene 3
CB-839: Telaglenastat (Glutaminase Inhibitor)
IL-2: Interleukin-2
IL-15: Interleukin-15
CD20: Cluster of Differentiation 20
GVHD: Graft-versus-Host Disease
GVL: Graft-versus-Leukemia
ROR1: Receptor Tyrosine Kinase-like Orphan Receptor 1
HIV: Human Immunodeficiency Virus
IL-21: Interleukin-21
CD27: Cluster of Differentiation 27
GMP: Good Manufacturing Practice
ACT: Adoptive Cell Transfer
KO: Knockout
TCR: T Cell Receptor
HMBPP: Hydroxy-Methyl-Butenyl Pyrophosphate
SDHA: Succinate Dehydrogenase Complex Flavoprotein Subunit A.
